# First case report of infection due to *Cupriavidus gilardii* in a patient without immunodeficiency: a case report

**DOI:** 10.1186/s12879-016-1838-y

**Published:** 2016-09-19

**Authors:** Takehito Kobayashi, Itaru Nakamura, Hiroaki Fujita, Ayaka Tsukimori, Akihiro Sato, Shinji Fukushima, Kiyofumi Ohkusu, Tetsuya Matsumoto

**Affiliations:** 1 Department of Infection Prevention and Control, Tokyo Medical University Hospital, 6-7-1 Nishishinjuku, Shinjuku-ku, Tokyo, 160-0023 Japan; 2 Department of Microbiology, Tokyo Medical University, 6-1-1 Shinjuku, Shinjuku-ku, Tokyo, 160-8402 Japan

**Keywords:** *Cupriavidus gilardii*, Glucose non-fermenting gram-negative rod, Sequencing analysis of the 16S rRNA gene, Pacemaker-associated bloodstream infection, Immunocompetent patient, Antimicrobial resistance, Case report

## Abstract

**Background:**

*Cupriavidus gilardii* is an aerobic, Gram-negative, glucose-nonfermenting rod that was first identified in 1999. Because of the difficulty in accurate species identification of *C. gilardii*, there are few case reports of infection caused by this organism. In previous reports, *C. gilardii* has been characterized as an organism with low pathogenicity that causes opportunistic infections.

**Case presentation:**

We encountered a case of pacemaker-associated bloodstream infection caused by *C. gilardii* in a 90-year old woman without obvious immunodeficiency*.* We identified the isolates as *C. gilardii* by sequencing of the 16S rRNA gene. The patient was treated with removal of the lead and administration of antimicrobial agents. Because of the acquisition of antibiotic resistance during antibiotic treatment, the antimicrobial agent was changed during the course of treatment.

**Conclusions:**

To our knowledge, this is the first report of an infection caused by this organism in a patient without obvious immunodeficiency. Although the true pathogenicity of *C. gilardii* is unclear, the possibility that it exerts pathogenicity not only in persons with immunodeficiency but also in immunocompetent persons is suggested.

## Background

*Cupriavidus gilardii* is an aerobic, Gram-negative, glucose-nonfermenting rod that was first identified in 1999 by Coenye et al. [1]. The history of taxonomy of this species is complicated; the species has been known by various names to date, including *Ralstonia gilardii*, *Wautersia gilardii,* and *C. gilardii* [2]. *C. gilardii* has been isolated from a wide variety of ecological niches, including plants and soils contaminated with heavy metals. This species has sporadically been isolated also from human clinical samples, including cerebrospinal fluid, bone marrow, wounds, furuncle, and the respiratory tract, but no information on its clinical importance has been reported to date [1, 3, 4]. In addition, *C. gilardii* has been identified in respiratory secretions of cystic fibrosis patients, but its clinical role has remained unclear [3].

Because of the difficulty in accurate species identification of *C. gilardii*, there are few case reports of infection caused by this organism. Only three clinical infections (bacteremia, catheter-associated sepsis, and muscular abscess) caused by *C. gilardii* have been reported to date [5–7]. Because all of the patients in these reports were severely immunocompromised, with diseases such as acute lymphocytic leukemia [5], severe idiopathic aplastic anemia [6], and renal transplantation [7], in previous reports, *C. gilardii* has been characterized as an organism with low pathogenicity that causes opportunistic infections.

## Case presentation

A 90-year old woman who had a pacemaker implanted 22 years previously for sick sinus syndrome and then had the pacemaker replaced one month previously was admitted to the community hospital owing to pus discharge from the wound. She had no other past medical history and did not have obvious immunodeficiency.

Because she had low grade fever with pus discharge from the wound, which suggested pacemaker infection, the main body of the pacemaker was removed, but the lead could not be removed. She underwent debridement of the wound and was administered antibiotics (ampicillin-sulbactam, cefepime [CFPM], and ciprofloxacin [CPFX]), but she was transferred to our hospital because of continued low-grade fever and high C-reactive protein level. Glucose non-fermentative Gram-negative rods (GNFR) of unidentified strain were isolated from the wound and blood culture. The isolates showed susceptibility to CPFX.

After the patient was transferred to our hospital, CPFX was administered continuously, but her low-grade fever persisted. Transthoracic echocardiography was performed, but there were no findings suggesting infectious endocarditis due to the pacemaker lead. Ampicillin-sulbactam was added from hospital day 9, but her fever did not decrease. Therefore, removal of the lead was attempted on hospital day 14 and part of the lead was cut. The same GNFR were isolated from the lead. Bacterial identification using ID test NF-18 (Nissui Pharmaceutical Co., Ltd., Tokyo, Japan) did not yield a definitive identification. The MicroScan WalkAway-96 plus system (Siemens Healthcare Diagnostics, Tokyo, Japan) also failed to identify the organism.

After cutting the lead, the patient’s body temperature decreased gradually. However, because of the low susceptibility to CPFX of the bacteria isolated from the lead, the antimicrobial agent was changed to CFPM from hospital day 18. Thereafter, although she occasionally had a low-grade fever, the patient’s poor general condition, including anorexia and malaise, gradually improved. From hospital day 31, the antimicrobial agent was changed to oral administration of minocycline, and she was discharged on hospital day 41.

### Microbiological analysis

The bacterial isolates appeared as very small colonies (<1 mm in diameter) on 5 % sheep blood agar (Nissui, Tokyo, Japan) after incubation at 24 h in normal atmosphere at 35 °C. On Gram staining, the organism was shown to be poorly stained, medium-sized gram-negative rods. Positive reactions were observed for oxidase activity and citrate utilization. Negative reactions were observed for the acidification of glucose, xylose, mannitol, maltose, sucrose, fructose, galactose, and lactose, urease production, nitrite reduction, hydrolysis of esculin and gelatin, arginine dihydrolase, ornithine decarboxylase, lysine decarboxylase, indole production, and hydrolysis of *o*-nitrophenyl-β-D-galactopyranoside (ID test NF-18; Nissui Pharmaceutical Co., Ltd., Tokyo, Japan). The production of pigment was not observed.

Antimicrobial susceptibility testing was performed according to the Clinical and Laboratory Standards Institute (CLSI) standards [8]. The minimum inhibitory concentrations of the antibiotic agents were determined by the broth microdilution method using MicroScan (Siemens, Tokyo, Japan). The breakpoints (susceptible, intermediate, or resistant) was determined according to *Pseudomonas aeruginosa* M100-S26 provided by CLSI [8].

In addition, we performed molecular identification by PCR amplification and sequencing analysis of the 16S rRNA gene using DNA extracted from the isolates. The universal primers 8UA (5′-AGAGTTTGATCMTGGCTCAG-3′) and 1485B (5′-ACGGGCGGTGTGTRC-3′) were used as described previously [9]. Sequencing analysis was performed using a GenBank BLAST search and EzTaxon (http://www.ezbiocloud.net/eztaxon/).

The susceptibility of the bacteria to various antimicrobial agents is shown in Table 1. The bacterial strain was identified as *C. gilardii* according to sequencing analysis of the 16S rRNA gene using DNA extracted from the isolates. The sequence of the 16S rRNA gene was 99.8 % identical (1,408 bp over the entire 1,411 bp fragment) with that of the type strain *C. gilardii* (ATCC 700815, accession number LMG 5886).

**Table 1:** Antimicrobial agent susceptibilities of *Cupriavidus gilardii*

Antimicrobial agents	Interpretation (MIC^※^)		
	Wound	Blood	Lead
PIPC	S (≤16)	R (>64)	S (≤8)
PIPC/TAZ	S (≤16)	R (>64)	S (≤8)
CAZ	R (>16)	R (>16)	R (>16)
AZT	–	R (>16)	R (>16)
CFPM	S (≤8)	S (<4)	S (≤4)
IPM/CS	–	I (4)	S (≤1)
MEPM	R (>8)	R (>8)	R(8)
GM	–	R (>8)	R (>8)
AMK	R (>32)	I (32)	I (32)
MINO	S (≤4)	S (<2)	S (≤2)
LVFX	–	S (<0.5)	S (2)
CPFX	S (≤1)	S (1)	R (>2)
ST	–	S (<2)	S (≤2)

*MIC* minimal inhibitory concentration, *PIPC* piperacillin, *PIPC/TAZ* piperacillin/tazobactam, *CAZ* ceftadizime, *AZT* aztreonam, *CFPM* cefepime, *IPM/CS* imipenem/cilastatin, *MEPM* meropenem, *GM* gentamicin, *AMK* amikacin, *MINO* minocycline, *LVFX* levofloxacin, *CPFX* ciprofloxacin, *ST* sulfamethoxazole/trimetoprim, ^※^: μg/mL, –: not tested

## Discussion

We identified the isolates as *C. gilardii* by sequencing of the 16S rRNA gene; however, identification of this strain is difficult using typical identification methods. In addition, there are no reports of identification of this strain using Matrix-Assisted Laser Desorption/Ionization Time of Flight (MALDI-TOF). If an isolate is found to be GNFR, appears as colonies, and has the biochemical characteristics described above, it may be necessary to suspect *C. gilardii*. Because of the difficulty in accurate species identification of *C. gilardii*, there are few case reports of infection caused by this organism. In previous reports, *C. gilardii* has been characterized as an organism with low pathogenicity that causes opportunistic infections. However, the patient in our present case did not have obvious immunodeficiency. To our knowledge, the presented case represents the first clinical infection due to *C. gilardii* in a patient without obvious immunodeficiency. This suggests that *C. gilardii* may cause infections in immunocompetent persons and has been underdiagnosed because of its difficulty of identification. In the previous case reports of infection by *C. gilardii*, the patients were relatively young (7, 12 and 36 years old) [5–7]. On the other hand, the patient of our case was elderly (90 years old). Although she had no obvious immunodeficiency, her elderly age might have affected the expression of pathogenicity of *C. gilardii*. In addition, it is probable that implantation of the pacemaker promoted the colonization of the organism and the development of infection. It is suspected that the portal of entry in this case was contamination of operation wound, but the origin of the organism is unclear. The patient did not have any history of contact with soils and plants.

*C. gilardii* has intrinsic resistance to many antimicrobial agents. In past reports and the present case, the isolates were often resistant to aztreonam, meropenem, gentamicin, and tobramycin. On the other hand, they were often susceptible to trimethoprim-sulfamethoxazole and tetracycline [5–7]. The optimal therapeutic regimen for treating infections caused by *C. gilardii* remains unclear because of the limited data. In addition, this organism was reported to have the ability to acquire resistance to antibiotics upon their continuous administration [6]. These characteristics of *C. gilardii* limit the effectiveness of antimicrobial agents and sometimes result in fatal outcomes [6]. In our case, we were forced to change the antibiotics being administered, because of the acquisition of resistance to CPFX. In a past case, the acquisition of resistance to CFPM and CPFX has been reported [6]. Although isolates in past reports and the present case were comparatively sensitive to fluoroquinolones, it may be necessary to pay attention to the progression of resistance during the administration of antibiotics. In the treatment of infections by *C. gilardii*, if the therapeutic response is reduced, it is necessary to reverify the antimicrobial sensitivity of the organism. In our case, difference in susceptibility for piperacillin and piperacillin-tazobactam in blood isolates and wound and lead isolates was observed. The cause of variability is unclear, but it has been reported that difference in susceptibility for piperacillin-tazobactam in isolates from the same patient was observed in previous report [6].

## Conclusions

The present case represents the fourth identified infection caused by *C. gilardii*. In addition, this is the first report of an infection of *C. gilardii* in a patient without obvious immunodeficiency. Although the true pathogenicity of *C. gilardii* is unclear, the possibility that it exerts pathogenicity not only in persons with immunodeficiency but also in immunocompetent persons is suggested. In addition, intrinsic antimicrobial resistance and the ability to acquire resistance to other antimicrobial agents were observed in isolates from the present patient as well as previous reports. For understanding the nature of *C. gilardii*, the accumulation of further cases is needed.

## Abbreviations

CFPM: Cefepime
CLSI: Clinical and Laboratory Standards Institute
CPFX: Ciprofloxacin
GNFR: Glucose non-fermentative gram-negative rods
